# Insomnia moderates the association between psychotic-like experiences and suicidal ideation in a non-clinical population: a network analysis

**DOI:** 10.1007/s00406-023-01653-3

**Published:** 2023-07-30

**Authors:** Błażej Misiak, Łukasz Gawęda, Ahmed A. Moustafa, Jerzy Samochowiec

**Affiliations:** 1 Department of Psychiatry, Wroclaw Medical University, Pasteura 10 Street, 50-367 Wroclaw, Poland; 2 Experimental Psychopathology Lab, Institute of Psychology, Polish Academy of Sciences, Warsaw, Poland; 3 School of Psychology & Centre for Data Analytics, Faculty of Society and Design, Bond University, Gold Coast, QLD Australia; 4 Department of Human Anatomy and Physiology, The Faculty of Health Sciences, University of Johannesburg, Johannesburg, South Africa; 5 Department of Psychiatry, Wroclaw Medical University, Wroclaw, Poland

**Keywords:** Psychosis, Suicide, Sleep, Early intervention

## Abstract

Psychotic-like experiences (PLEs) have been associated with poor sleep quality and increased suicide risk. However, the association between PLEs, insomnia and suicide risk has not been thoroughly investigated in prior studies. In this study, we aimed to explore as to whether insomnia moderates the association between PLEs and suicidal ideation. The study was performed in 4203 young adults (aged 18–35 years, 63.8% females). Data were collected using self-reports. Moderation analysis demonstrated that PLEs are associated with higher levels of the current suicidal ideation only in participants with greater severity of insomnia (*B* = 0.003, *p* < 0.001). This analysis included age, gender, education, occupation and depressive symptoms as covariates. Moreover, the network analysis demonstrated that nodes representing PLEs are connected to the node of current suicidal ideation only in participants with greater severity of insomnia. The nodes of PLEs connected to the current suicidal ideation node captured PLEs representing deja vu experiences, auditory hallucination-like experiences and paranoia (edge weights between 0.011 and 0.083). Furthermore, nodes representing PLEs were the three most central nodes in the network analysis of individuals with higher levels of insomnia (strength centrality between 0.96 and 1.10). In turn, the three most central nodes were represented by depressive symptoms in the network analysis of individuals with lower levels of insomnia (strength centrality between 0.67 and 0.79). Findings from this study indicate that insomnia might be an important risk factor of suicide in people with PLEs, especially those reporting deja vu experiences, auditory hallucination-like experiences and paranoia.

**Supplementary Information:**

The online version contains supplementary material available at 10.1007/s00406-023-01653-3.

## Introduction

Psychotic-like experiences (PLEs) capture a range of subclinical phenomena that cannot be the basis for diagnosing mental disorders due to insufficient severity or impact on general functioning. The lifetime prevalence of PLEs has been estimated at 5.8% of the general population; however, it might be higher in younger people [1, 2]. It has been shown that PLEs do not form a discrete categorical construct but are distributed dimensionally in non-clinical populations and high-risk individuals [3, 4]. Notably, PLEs are not the predictors specific for the development of psychosis, but are associated with a variety of mental health outcomes [5]. Therefore, investigating PLEs, as substrates of subclinical psychopathology, might be informative for interventions targeting preclinical stages of mental disorders.

It is important to note that PLEs cover a range of delusion-like and hallucination-like phenomena that are known to predict the development of various mental disorders that fall beyond the psychosis spectrum [5–7]. Factor analyses of tools that record the presence of PLEs also show that they do not form a homogenous construct [8]. In this regard, the approach based on the assumption that a common latent disorder or mechanism underlies the development and consequences of PLEs might be insufficient. A novel approach, i.e., the network analysis assumes that psychopathology covers the systems of causally associated symptoms rather than the effects of a latent disorder or mechanism [9–11]. The network analysis has already been applied by studies that aimed to understand the phenomenology of PLEs and their associations with risk and protective factors as well as other domains of psychopathology. For instance, it has been found that PLEs are associated with a variety of social and behavioral problems, suicidal behavior, and depressive symptoms in adolescents [12]. Another study demonstrated the importance of social contexts in understanding the consequences of PLEs [13]. The authors of this study found that PLEs are significantly less interconnected and show weaker associations with the level of distress in populations representing low- and middle-income countries compared to those from high-income countries.

Although PLEs are subclinical phenomena, it has been shown that these experiences are significantly associated with the risk of suicidal thoughts, plans and attempts [14–16]. However, specific PLEs might be differentially associated with a risk of suicidal ideation and behaviors with particularly strong associations reported for thought control, auditory hallucinations, suspiciousness, and nihilistic thinking/dissociative experiences [17]. Moreover, little is known about processes that moderate the association between PLEs and suicide risk. For instance, it has been found that greater impulsivity and emotion dysregulation make individuals with PLEs more prone to develop suicidal ideation and behaviors [18].

There is accumulating evidence that PLEs are associated with poor sleep quality [19–21]. Studies based on longitudinal designs and experience sampling methodology suggest that there might be a bidirectional association between poor sleep quality and PLEs [19, 22, 23]. It has also been demonstrated that cognitive-behavioral therapy of insomnia might reduce the level of paranoia and hallucinations in university students [24]. Moreover, experimental studies show that sleep deprivation initiates the onset of PLEs, and that PLEs are reduced after restoring sleep schedules [25, 26]. Taken together, these observations suggest a vicious cycle conceptualization in which sleep disturbance gives rise to PLEs that might in turn contribute to increased distress further enhancing poor sleep quality [27]. Importantly, there is evidence that sleep disturbance and PLEs might share overlapping neural mechanisms represented by a reduction in volumes of the left thalamus as recently reported [28].

As similar to PLEs, sleep disturbance might be associated with increased risk of suicide. For instance, a recent meta-analysis of cohort studies revealed that sleep disturbance is associated with over threefold higher incident risk of suicide attempt and almost twofold higher incident risk of completed suicide [29]. Another meta-analysis of sleep measures demonstrated that decreased total sleep time is related to current suicidal behaviors [30]. However, little is known about the interaction between sleep disturbance and PLEs in impacting suicide risk. To date, only one study tested this effect in help-seeking individuals [31]. The authors observed that PLEs are associated with suicidal ideation only at higher levels of sleep difficulties. Nevertheless, this study did not investigate which specific PLEs contribute to suicidal ideation at higher levels of sleep disturbance. In this regard, we aimed to extend these findings over a larger sample of non-clinical young adults. Specifically, the aim of our study was to test the moderating effect of insomnia on the association between PLEs and the current suicidal ideation. Additionally, we explored which PLEs are related to the current suicidal ideation at various levels of insomnia in the network analysis.

## Methods

### Participants

Participants were enrolled by means of the snowball sampling method administered in social media and surveying websites. They were assessed through the computer assisted web interview (CAWI) implemented between April and October, 2022. All of them were informed about anonymous character and confidentiality of the survey. Inclusion criteria were as follows: age between 18 and 35 years and a negative history of psychiatric treatment. Participants assessed in the present study were individuals screened for PLEs in the first part of a bigger project examining epigenetic mechanisms of psychosis proneness. The study received approval of the Ethics Committees at the Institute of Psychology (Polish Academy of Sciences in Warsaw, Poland, approval number: 16/VII/2022), Wroclaw Medical University (Wroclaw, Poland, approval number: 129/2022) and Pomeranian Medical University (Szczecin, Poland, approval number: KB-006/25/2022).

### Assessments

#### PLEs

Assessment of PLEs was based on a 16-item questionnaire (the content of specific items is provided in Supplementary Table 1). It was developed to record the presence of PLEs during the preceding month. All items were based on a four-point scale (1—“never”; 2—“sometimes”; 3—“often” and 4—“almost always”), and participants were asked to score experiences that cannot be attributed to substance use. The items were obtained from the following questionnaires: (1) the Revised Hallucination Scale (RHS) [32–34] (3 items); (2) the Revised Green et al. Paranoid Thoughts Scale (GPTS) [35] (4 items) and 3) the Prodromal Questionnaire–16 (PQ–16) [36] (9 items). It has been shown that the PQ–16 is a valid tool in screening for psychosis risk states [36]. Also, there is evidence that the GPTS has good psychometric properties, including test–retest reliability, convergent and construct validity, and sensitivity to change [37–39]. The revised GPTS is a more precise measure with excellent psychometric properties [35]. Similarly, it has been shown that the RHS is characterized by good psychometric properties in terms of test–retest reliability as well as convergent and construct validity [34]. We decided to combine items from different questionnaires as available tools often measure the occurrence of heterogenous experiences, including delusion-like experiences, hallucination-like experiences, negative, and depressive symptoms [40]. The total score of the questionnaire ranges between 16 and 64 points (higher scores represent higher level of PLEs). In the present study, the Cronbach’s alpha and the McDonald’s omega were 0.811 and 0.810, respectively.

#### Insomnia

Four items from the Insomnia Severity Index (ISI) were administered to record insomnia [41]. Three items were used to assess difficulty in falling asleep, difficulty staying asleep and problems with waking too early (a 5-point scale scored from 0—“none” to 4—“very severe). The fourth item is about the level of satisfaction/dissatisfaction from current sleep (a 5-point scale scored from 0—“very satisfied” to 4—“very dissatisfied”). The total score of this questionnaire ranges between 0 and 16 (higher scores are indicative of higher insomnia severity). It has been shown that the ISI is a valid and reliable instrument to quantify perceived severity of insomnia [42]. The Cronbach’s alpha and the McDonald’s omega of the insomnia questionnaire were 0.721 and 0.723, respectively.

#### Depressive symptoms and the current suicidal ideation

To assess the occurrence of depressive symptoms and suicidal ideation, we used the Patient Health Questionnaire-9 (PHQ-9). It consists of nine items that measure the presence of depressive symptoms over the preceding 2 weeks (a four-point scale with responses from 0—“not at all” to 3—“nearly every day”). The last PHQ-9 item (“thoughts that you would be better off dead, or of hurting yourself in some way?”) was used to assess the current suicidal ideation. It has been demonstrated that the PHQ-9 is a valid and reliable tool to screen for depression in various populations [43]. In the present study, the Cronbach’s alpha and the McDonald’s omega of the PHQ-9 were 0.810 (the same value for both coefficients).

### Data analysis

Only complete data were analyzed (*n* = 4200). Correlations between continuous variables (scores of PLEs, depressive symptoms and insomnia) were analyzed using the Spearman rank correlation coefficients. Moderation was tested in the PROCESS macro (Model 1) [44]. The scores of PLEs were included as a predictor (X variable), while suicidal ideation (the PHQ-9 item 9 score) was included as the outcome variable (Y variables). The total score of insomnia was included as a moderator (moderator variable W). Age, gender, educational level, occupation and depressive symptoms (total scores from PHQ-9 items 1–8) were added as covariates. The Johnson-Neyman technique was used to identify the range of insomnia scores for which the interaction effect is significant. Results were interpreted as significant if the p-value was lower than 0.05.

We divided participants into two groups based on the level of insomnia indicated according to the Johnson-Neyman technique. Subsequently, a separate network analyses were carried out in both groups using the R software. We used the same variables as those included in the moderation analysis. The Mixed Graphical Models, implemented in the *mgm* package [45], were used as binary (gender, education, and occupation) and continuous variables were included (depressive symptoms and PLEs). To improve prediction accuracy and interpretability of results, the L1-penalized regression (LASSO) was implemented. The LASSO reduces the number of estimated parameters to avoid spurious associations by shrinking partial correlation coefficients. The penalty parameter was selected by the Extended Bayesian Information Criterion (EBIC) using the tuning parameter λ that controls the level of sparsity [46]. The λ parameter was set at 0.5 in the current study [47].

The network included sociodemographic characteristics, PLEs and depressive symptoms (nodes) that are connected with edges. The edge thickness indicates the strength of the association between nodes (i.e. thicker nodes reflect stronger associations). The centrality of nodes was analyzed by calculating the node strength. The node strength is the most commonly used indicator of centrality and reflects the sum of all edge weights connected to the node [47–49]. Moreover, the node predictability was calculated. The node predictability can be defined as the proportion of variance explained by nodes directly connected to a specific node. The visualization of results was performed using the *qgraph* package [50].

Finally, the network accuracy and stability were analyzed in the *bootnet* package [47]. The bootstrapping was carried out with 1000 iterations in order to analyze the stability of the node strength. The stability of the node strength was visualized and calculated using the correlation stability coefficient (CS-C) that needs to be higher than 0.25. Moreover, the 95% confidence interval (CI) of edge weights was analyzed using the non-parametric bootstrapping with 1000 iterations. A greater 95%CI corresponds to lower precision in the estimation of edge weights.

## Results

### Participants

The sample characteristics are shown in Table 1. A total of 4203 participants were enrolled (aged 25.3 ± 5.7 years, 63.8% females). Most of them were students (50.7%) and individuals with a high level of education (40.6%).

**Table 1:** Descriptive characteristics of the sample

	Mean ± SD or *n* (%)
Age, years	25.3 ± 5.7
Gender, females	2680 (63.8)
Education	
Primary	142 (3.5)
Vocational	64 (1.5)
Secondary	1271 (30.2)
Incomplete higher	1018 (24.2)
Higher	1708 (40.6)
Occupation	
Unemployed	169 (4.0)
Student	2129 (50.7)
Employed	1864 (44.3)
Rent	41 (1.0)
Family history of schizophrenia	229 (5.4)
Family history of depression and bipolar disorder	928 (22.1)
PLEs	22.4 ± 5.1^a^
Depressive symptoms	9.5 ± 5.6^b^
Insomnia	5.7 ± 3.7
Suicidal ideation	0.4 ± 0.8^a^
Suicidal ideation, yes	1214 (28.9)^a^

*PLEs* psychotic-like experiences

^a^One case with missing data

^b^Two cases with missing data

### Moderation analyses

**Fig. 1:**
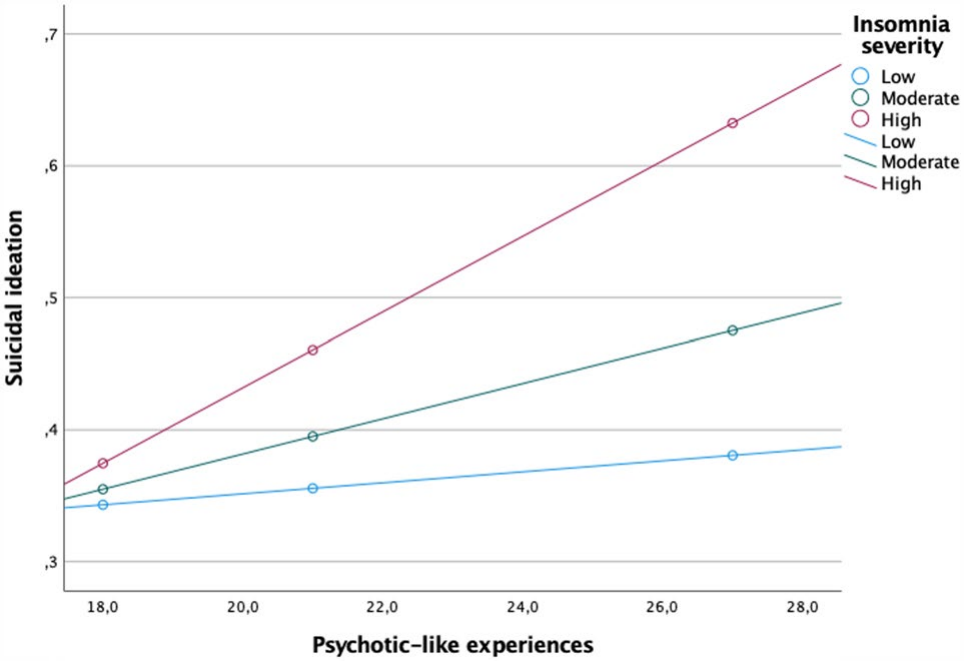
Correlations of the scores of PLEs with the suicidal ideation score at various levels of insomnia

All measures tested in the present study were significantly and positively correlated (Table 2). Results of moderation analysis are reported in Table 3. There were significant positive associations of insomnia, the insomnia × PLEs interaction, depressive symptoms, education and occupation with the current suicidal ideation. The model explained 27.1% of variance in the current suicidal ideation (*R*^2^ = 0.271). Adding the interaction term to the model was associated with a significant *R*^2^ change (*R*^2^ change = 0.006, *F* = 37.274, *p* < 0.001). The insomnia score defining the Johnson-Neyman region of significance was 2.926 (% of non-significant correlations below the cut-off = 20.114 and % of significant correlations above the cut-off = 79.886). Conditional effects of the focal predictor (scores of PLEs) at values of the moderator (insomnia scores) are shown in Fig. 1. Regression lines were plotted for the following levels of insomnia: (1) low level = 2.0 (*B* = 0.004, *SE* = 0.004, *t* = 1.065, *p* = 0.287, 95%CI − 0.004–0.012); (2) moderate level = 5.0 (B = 0.013, *SE* = 0.003, *t* = 4.562, *p* < 0.001, 95%CI 0.008–0.019) and (3) high level = 10.0 (*B* = 0.029, *SE* = 0.003, *t* = 4.562, *p* < 0.001, 95%CI 0.023–0.034).

**Table 2:** Bivariate correlations

	1	2	3
1. PLEs	–		
2. Depressive symptoms	*r* = 0.479	–	
3. Insomnia	*r* = 0.384	*r* = 0.480	–
4. Suicidal ideation	*r* = 0.326	*r* = 0.478	*r* = 0.313

*PLEs* psychotic-like experiences

*p* < 0.001 for all correlations

**Table 3:** Results of the moderation analysis

	B	SE	*t*	*p*	95%CI
PLEs	− 0.002	0.005	− 0.414	0.679	− 0.001 to 0.007
Insomnia	0.051	0.012	4.319	** < 0.001**	0.028 to 0.074
PLEs × insomnia	0.003	0.001	6.105	** < 0.001**	0.002 to 0.004
Depressive symptoms	0.062	0.003	23.431	** < 0.001**	0.057 to 0.067
Age	0.001	0.002	0.143	0.886	− 0.004 to 0.004
Gender	− 0.014	0.023	− 0.601	0.548	− 0.058 to 0.031
Education	− 0.050	0.023	− 2.137	**0.033**	− 0.096 to − 0.004
Occupation	− 0.140	0.050	− 2.786	**0.005**	− 0.239 to − 0.042

Significant association (*p* < 0.05) are marked in bold

### Network analyses

#### Network structure

**Fig. 2:**
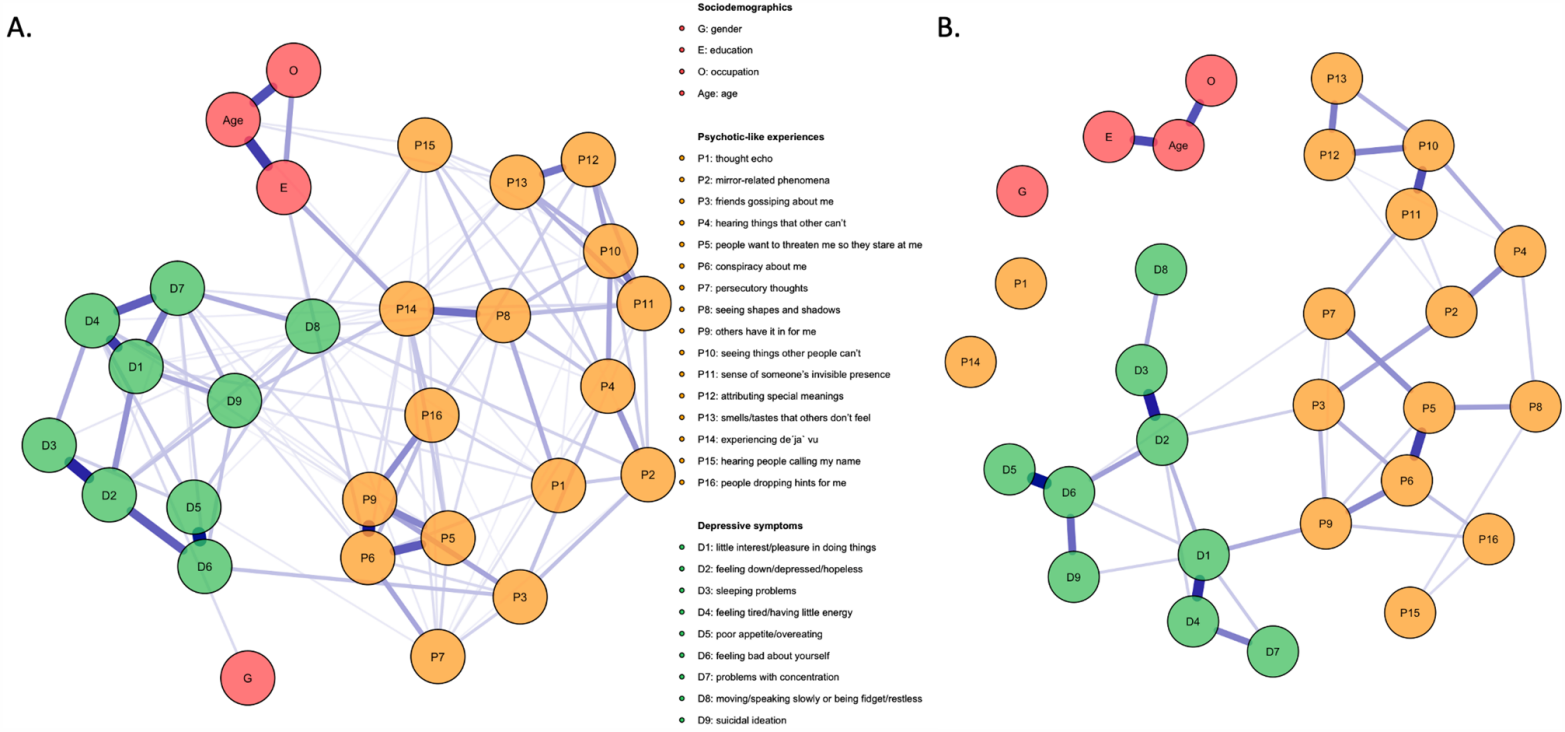
The networks analyzed in the present study. **A** refers to the network analysis of participants with insomnia, while **B** shows the network analysis in participants without insomnia

The networks analyzed in the present study are shown in Fig. 2. The network shown in Fig. 2A refers to participants with the ISI scores higher than 2 (here and after referred to as individuals with insomnia, *n* = 3352), while Fig. 2B refers to participants with the ISI scores lower than 3 (here and after referred to as individuals without insomnia, *n* = 848). The cut-off was established based on the Johnson-Neyman region. No negative nodes were found. In the network of participants with insomnia, all nodes appeared to be well connected, while in the network of participants without insomnia some nodes were isolated (i.e., the nodes of sociodemographic characteristics, P1–“thought echo”, and P14–“deja vu experiences”.

Edge weights are shown in Supplementary Tables 2, 3. The following nodes of PLEs (in the descending order of edge weights) were connected to the node of suicidal ideation (D9) in participants with insomnia: “deja vu experiences” (P14, edge weight = 0.083), “conspiracy about me” (P6, edge weight = 0.045), “thought echo” (P1, edge weight = 0.042), “people want to threaten me so they stare at me” (P5, edge weight = 0.030), “others have it in for me” (P9, edge weight = 0.024), and “hearing people calling my name” (P15, edge weight = 0.011). The P14–D9 edge weight was significantly higher than the P5–D9, P9–D9 and P15–D9 edge weights (Supplementary Figs. 1, 2). Importantly, there were no direct connections between nodes of PLEs and the D9 node in participants without insomnia.

#### Central nodes

Strength centrality values and their comparison are plotted in Supplementary Figs. 3, 4. The three most central nodes in participants with insomnia included “conspiracy about me” (P6, strength = 1.10), “deja vu experiences” (P14, strength = 1.00), and “others have it in for me” (P9, strength = 0.96). In turn, in participants without insomnia these nodes were represented by “feeling bad about yourself” (D6, strength = 0.79), “little interest/pleasure in doing things” (D1, strength = 0.70), and “feeling down/depressed/hopeless” (D2, strength = 0.67).

#### Node predictability

The mean predictability of the network was 0.241 (i.e., the mean variance of each node explained by nodes directly connected to it was 24.1%) in the analysis of participants with insomnia and 0.165 (i.e., the mean variance of each node explained by nodes directly connected to it was 16.5%) in the analysis of participants without insomnia (Supplementary Table 4). In the analysis of participants with insomnia, the mean predictability of specific groups of nodes was 0.248 for PLEs and 0.319 for depressive symptoms. In turn, in the analysis of participants without insomnia, the mean predictability of specific groups of nodes was 0.159 for PLEs and 0.242 for depressive symptoms. In participants with insomnia, the node with the highest predictability was “conspiracy about me” (P6, predictability = 0.452) while the node with the lowest predictability was “hearing people calling my name” (P15, predictability = 0.126). In participants without insomnia, the node with the highest predictability was represented by “feeling bad about yourself” (D6, predictability = 0.387), while the node with the lowest predictability was “thought echo” (P1, predictability = 0).

#### Network stability and accuracy

The node-specific strength appeared to be stable when dropping various proportions of data (Supplementary Fig. 5). The CS-C value was 0.75 (the same values for edges and strength values) in the analysis of participants with insomnia. In turn, in the analysis of participants without insomnia, the CS-C values were 0.59 (for edges) and 0.36 (for strength values). These estimates indicate that the network models were robust. The majority of bootstrapped 95%CI ranges of edge weights were relatively narrow indicating sufficient accuracy (Supplementary Fig. 6).

## Discussion

Findings from our study indicate that PLEs are associated with the current suicide risk only in subjects with insomnia after controlling for the effects of sociodemographic characteristics and depressive symptoms. These findings are in agreement with those obtained by Thompson et al. [31], who also found a moderating effect of sleep quality on the association between PLEs and suicidal ideation in help-seeking individuals. Our findings extend these observations over the non-clinical sample of individuals with less severe psychopathological symptoms. Moreover, a recent study performed in college students demonstrated that previous suicidal ideation and low subjective quality of sleep are the most robust predictors of the current suicidal ideation [51]. Other factors associated with higher risk of the current suicidal ideation in this sample included paranoid thoughts, internet addiction, poor self-rated physical health, poor self-rated overall health, emotional abuse, low average annual household income per person and heavy study pressure.

Another important observation from this study is that deja vu experiences, auditory hallucination-like experiences and paranoia might be most closely related to suicidal ideation in subjects with PLEs reporting insomnia. No connections between nodes of PLEs and the node of current suicidal ideation were found in participants without insomnia. Further, nodes representing paranoia and deja vu experiences represented the three most central nodes in the network analysis of participants with insomnia. Also, the item that captured paranoia (“conspiracy about me”) had the highest predictability in participants with insomnia. In turn, depressive symptoms were the three most central nodes in the network analysis of participants without insomnia. The PHQ-9 item 6 (“feeling bad about yourself”) had the highest predictability in this subgroup of participants. The connection between deja vu experiences and the current suicidal ideation had the largest edge weight among connections between PLEs and the current suicidal ideation. The phenomenological position of deja vu experiences remains unclear. However, some authors classify deja vu experiences among dissociation symptoms [52]. It has been found that dissociation might be a transdiagnostic risk factor of suicide [53]. In general, our findings are also similar to those obtained by Jay et al. [17], who demonstrated that thought control, auditory hallucinations, suspiciousness, and nihilistic thinking/dissociative experiences represent PLEs that are the most strongly associated with suicidal ideation among children with PLEs. Moreover, paranoia has been repeatedly associated with higher risk of suicide in clinical and non-clinical samples [54–56].

Our findings should be interpreted with caution due to certain limitations. We did not use any standardized tools to assess psychiatric diagnosis in participants. Therefore, translation of findings into clinical practice should be approached cautiously. However, there is evidence that PLEs represent a transdiagnostic risk factor of mental disorders [6, 7]. Moreover, self-reported PLEs have been found to predict the development of psychosis in epidemiological studies of non-help-seeking individuals [57]. Also, self-reported PLEs that have been found to represent false positive findings in standardized clinical assessment might predict the development of psychosis, mood and anxiety disorders as well as low social functioning [58, 59]. Another limitation is that the use of a snowball sampling methodology might be characterized by limited accuracy and low representativeness of participants [60]. Indeed, we were not able to record the number and data of participants who declined to participate in the survey. Moreover, causal associations cannot be indicated as the cross-sectional design was used. However, according to existing evidence in the field, bidirectional associations between PLEs and sleep disturbance are most likely to occur [19, 22, 23]. Also, all assessments were performed using self-reports with selected items derived from specific tools. Among them, suicidal ideation was assessed using only one item from the PHQ-9 (item 9). This item might also capture the current intent of non-suicidal self-injuries and does not cover all aspects of suicidality (e.g. lifetime occurrence of suicide attempts, ideations and plans). However, in the present study, we focused on the current psychopathology. Also, it has been shown that the PHQ-9 item 9 might hold usefulness in stratifying the risk of suicide [61–63]. At this point, it is also important to highlight the lack of objective measures for insomnia. Finally, it needs to be pointed out that results of the network analysis might depend on the inclusion of specific variables. In this regard, it should be noted that the association between PLEs and the current suicidal ideation reported in the present study might simply reflect shared correlates of PLEs and suicidality (e.g. childhood trauma, substance use, and other dimensions of psychopathology) [64].

In sum, observations from the present study provide certain translational perspectives that might be of importance in clinical practice. Specifically, our findings indicate that insomnia might be an important aspect in subjects with PLEs increasing the occurrence of suicidal ideation. From the phenomenological point of view, deja vu experiences, hallucination-like experiences and paranoia might be more closely related to suicidal ideation in subjects with PLEs who also report insomnia. Targeting sleep quality might be important for suicide prevention among individuals with PLEs. However, additional studies in clinical samples are needed to develop specific recommendations.

### Supplementary Information

Below is the link to the electronic supplementary material.

Supplementary file1 (DOCX 1788 KB)

## Data Availability

Data generated in the present study are available in the Open Science Framework (OSF) database (https://doi.org/10.17605/OSF.IO/8JU6B).

## References

[1] McGrath JJ, Saha S, Al-Hamzawi A, Alonso J, Bromet EJ, Bruffaerts R (2015). Psychotic experiences in the general population: a cross-national analysis based on 31,261 respondents from 18 countries. JAMA Psychiat.

[2] Kelleher I, Connor D, Clarke MC, Devlin N, Harley M, Cannon M (2012). Prevalence of psychotic symptoms in childhood and adolescence: a systematic review and meta-analysis of population-based studies. Psychol Med.

[3] Taylor MJ, Freeman D, Ronald A (2016). Dimensional psychotic experiences in adolescence: evidence from a taxometric study of a community-based sample. Psychiatry Res.

[4] Adjorlolo S, Anum A, Adjorlolo P (2021). Latent structure of psychotic-like experiences in adolescents: evidence from a multi-method taxometric study of a school-based sample in Ghana. Psychiatry Res.

[5] Lindgren M, Numminen L, Holm M, Therman S, Tuulio-Henriksson A (2022). Psychotic-like experiences of young adults in the general population predict mental disorders. Psychiatry Res.

[6] Staines L, Healy C, Coughlan H, Clarke M, Kelleher I, Cotter D (2022). Psychotic experiences in the general population, a review; definition, risk factors, outcomes and interventions. Psychol Med.

[7] McGorry PD, Hartmann JA, Spooner R, Nelson B (2018). Beyond the “at risk mental state” concept: transitioning to transdiagnostic psychiatry. World Psychiatry.

[8] Howie C, Hanna D, Shannon C, Davidson G, Mulholland C (2022). The structure of the prodromal questionnaire-16 (PQ-16): exploratory and confirmatory factor analyses in a general non-help-seeking population sample. Early Interv Psychiatry.

[9] Borsboom D, Cramer AO (2013). Network analysis: an integrative approach to the structure of psychopathology. Annu Rev Clin Psychol.

[10] Borsboom D (2017). A network theory of mental disorders. World Psychiatry.

[11] Jones PJ, Mair P, McNally RJ (2018). Visualizing psychological networks: a tutorial in R. Front Psychol.

[12] Fonseca-Pedrero E, Muniz J, Gacia-Portilla MP, Bobes J (2021). Network structure of psychotic-like experiences in adolescents: links with risk and protective factors. Early Interv Psychiatry.

[13] Wusten C, Schlier B, Jaya ES, Fonseca-Pedrero E, Genetic Risk and Outcome of Psychosis (GROUP) Investigators (2018). Psychotic experiences and related distress: a cross-national comparison and network analysis based on 7141 participants from 13 countries. Schizophr Bull.

[14] Jang JH, Lee YJ, Cho SJ, Cho IH, Shin NY, Kim SJ (2014). Psychotic-like experiences and their relationship to suicidal ideation in adolescents. Psychiatry Res.

[15] Gaweda L, Pionke R, Krezolek M, Frydecka D, Nelson B, Cechnicki A (2020). The interplay between childhood trauma, cognitive biases, psychotic-like experiences and depression and their additive impact on predicting lifetime suicidal behavior in young adults. Psychol Med.

[16] Nishida A, Shimodera S, Sasaki T, Richards M, Hatch SL, Yamasaki S (2014). Risk for suicidal problems in poor-help-seeking adolescents with psychotic-like experiences: findings from a cross-sectional survey of 16,131 adolescents. Schizophr Res.

[17] Jay SY, Schiffman J, Grattan R, O'Hare K, Klaunig M, DeVylder J (2022). A deeper dive into the relation between psychotic-like experiences and suicidal ideation and behaviors in children across the United States. Schizophr Bull.

[18] Grattan RE, Karcher NR, Maguire AM, Hatch B, Barch DM, Niendam TA (2021). Psychotic like experiences are associated with suicide ideation and behavior in 9 to 10 year old children in the United States. Res Child Adolesc Psychopathol.

[19] Zhou R, Foo JC, Yamaguchi S, Nishida A, Ogawa S, Usami S (2022). The longitudinal relationship between sleep length and psychotic-like experiences in adolescents. Psychiatry Res.

[20] Ered A, Cooper S, Ellman LM (2018). Sleep quality, psychological symptoms, and psychotic-like experiences. J Psychiatr Res.

[21] Korenic SA, Ered A, Pierce KM, Calvo EM, Olino TM, Murty VP (2021). Examining self-reported social functioning, sleep quality, and psychotic-like experiences in college students. J Psychiatr Res.

[22] Simor P, Bathori N, Nagy T, Polner B (2019). Poor sleep quality predicts psychotic-like symptoms: an experience sampling study in young adults with schizotypal traits. Acta Psychiatr Scand.

[23] Hennig T, Schlier B, Lincoln TM (2020). Sleep and psychotic symptoms: an actigraphy and diary study with young adults with low and elevated psychosis proneness. Schizophr Res.

[24] Freeman D, Sheaves B, Goodwin GM, Yu LM, Nickless A, Harrison PJ (2017). The effects of improving sleep on mental health (OASIS): a randomised controlled trial with mediation analysis. Lancet Psychiatry.

[25] Petrovsky N, Ettinger U, Hill A, Frenzel L, Meyhofer I, Wagner M (2014). Sleep deprivation disrupts prepulse inhibition and induces psychosis-like symptoms in healthy humans. J Neurosci.

[26] Orzel-Gryglewska J (2010). Consequences of sleep deprivation. Int J Occup Med Environ Health.

[27] Lunsford-Avery JR, Mittal VA (2013). Sleep dysfunction prior to the onset of schizophrenia: a review and neurodevelopmental diathesis-stress conceptualization. Clin Psychol Sci Pract.

[28] Lunsford-Avery JR, Damme KSF, Vargas T, Sweitzer MM, Mittal VA (2021). Psychotic-like experiences associated with sleep disturbance and brain volumes in youth: findings from the adolescent brain cognitive development study. JCPP Adv.

[29] Dong M, Lu L, Sha S, Zhang L, Zhang Q, Ungvari GS (2021). Sleep disturbances and the risk of incident suicidality: a systematic review and meta-analysis of cohort studies. Psychosom Med.

[30] Romier A, Maruani J, Lopez-Castroman J, Palagini L, Serafini G, Lejoyeux M (2023). Objective sleep markers of suicidal behaviors in patients with psychiatric disorders: a systematic review and meta-analysis. Sleep Med Rev.

[31] Thompson EC, Jay SY, Andorko ND, Millman ZB, Rouhakhtar PR, Sagun K (2021). Sleep quality moderates the association between psychotic-like experiences and suicidal ideation among help-seeking university students. Psychiatry Res.

[32] Gaweda L, Kokoszka A (2011). Polish version of the revised hallucination scale (RHS) by Morrison et al. Its factor analysis and the prevalence of hallucinatory-like experiences among healthy participants. Psychiatr Pol.

[33] Morrison AP, Wells A, Nothard S (2000). Cognitive factors in predisposition to auditory and visual hallucinations. Br J Clin Psychol.

[34] Morrison AP, Wells A, Nothard S (2002). Cognitive and emotional predictors of predisposition to hallucinations in non-patients. Br J Clin Psychol.

[35] Freeman D, Loe BS, Kingdon D, Startup H, Molodynski A, Rosebrock L (2021). The revised Green et al. Paranoid Thoughts Scale (R-GPTS): psychometric properties, severity ranges, and clinical cut-offs. Psychol Med.

[36] Ising HK, Veling W, Loewy RL, Rietveld MW, Rietdijk J, Dragt S (2012). The validity of the 16-item version of the prodromal questionnaire (PQ-16) to screen for ultra high risk of developing psychosis in the general help-seeking population. Schizophr Bull.

[37] Green CE, Freeman D, Kuipers E, Bebbington P, Fowler D, Dunn G (2008). Measuring ideas of persecution and social reference: the Green et al. Paranoid Thought Scales (GPTS). Psychol Med.

[38] Freeman D, Pugh K, Vorontsova N, Antley A, Slater M (2010). Testing the continuum of delusional beliefs: an experimental study using virtual reality. J Abnorm Psychol.

[39] Freeman D, Pugh K, Antley A, Slater M, Bebbington P, Gittins M (2008). Virtual reality study of paranoid thinking in the general population. Br J Psychiatry.

[40] Hinterbuchinger B, Mossaheb N (2021). Psychotic-like experiences: a challenge in definition and assessment. Front Psychiatry.

[41] Morin CM, Belleville G, Belanger L, Ivers H (2011). The Insomnia Severity Index: psychometric indicators to detect insomnia cases and evaluate treatment response. Sleep.

[42] Bastien CH, Vallieres A, Morin CM (2001). Validation of the insomnia severity index as an outcome measure for insomnia research. Sleep Med.

[43] Negeri ZF, Levis B, Sun Y, He C, Krishnan A, Wu Y (2021). Accuracy of the Patient Health Questionnaire-9 for screening to detect major depression: updated systematic review and individual participant data meta-analysis. BMJ.

[44] Hayes A (2018). Introduction to mediation, moderation, and conditional process analysis: a regression-based approach.

[45] Haslbeck JMB, Waldorp LJ (2020). MGM: estimating time-varying mixed graphical models in high-dimensional data. J Stat Softw.

[46] Foygel R, Drton M (2010). Extended Bayesian information criteria for Gaussian graphical models. Adv Neural Inf Process Syst.

[47] Epskamp S, Fried EI (2018). A tutorial on regularized partial correlation networks. Psychol Methods.

[48] Scott J, Crouse JJ, Ho N, Carpenter J, Martin N, Medland S (2021). Can network analysis of self-reported psychopathology shed light on the core phenomenology of bipolar disorders in adolescents and young adults?. Bipolar Disord.

[49] Fonseca-Pedrero E (2017). Network analysis: a new way of understanding psychopathology?. Rev Psiquiatr Salud Ment.

[50] Epskamp S, Cramer AOJ, Waldorp LJ, Schmittmann VD, Borsboom D (2012). Qgraph: network visualizations of relationships in psychometric data. J Stat Softw.

[51] Liao S, Wang Y, Zhou X, Zhao Q, Li X, Guo W (2022). Prediction of suicidal ideation among Chinese college students based on radial basis function neural network. Front Public Health.

[52] Brauer R, Harrow M, Tucker GJ (1970). Depersonalization phenomena in psychiatric patients. Br J Psychiatry.

[53] Calati R, Bensassi I, Courtet P (2017). The link between dissociation and both suicide attempts and non-suicidal self-injury: meta-analyses. Psychiatry Res.

[54] Lee E, Karim H, Andreescu C, Mizuno A, Aizenstein H, Lee H (2022). Network modeling of anxiety and psychological characteristics on suicidal behavior: cross-sectional study. J Affect Disord.

[55] Bird JC, Fergusson EC, Kirkham M, Shearn C, Teale AL, Carr L (2021). Paranoia in patients attending child and adolescent mental health services. Aust N Z J Psychiatry.

[56] Freeman D, Bold E, Chadwick E, Taylor KM, Collett N, Diamond R (2019). Suicidal ideation and behaviour in patients with persecutory delusions: prevalence, symptom associations, and psychological correlates. Compr Psychiatry.

[57] Kaymaz N, Drukker M, Lieb R, Wittchen HU, Werbeloff N, Weiser M (2012). Do subthreshold psychotic experiences predict clinical outcomes in unselected non-help-seeking population-based samples? A systematic review and meta-analysis, enriched with new results. Psychol Med.

[58] Bak M, Delespaul P, Hanssen M, de Graaf R, Vollebergh W, van Os J (2003). How false are “false” positive psychotic symptoms?. Schizophr Res.

[59] van der Steen Y, Myin-Germeys I, van Nierop M, Ten Have M, de Graaf R, van Dorsselaer S (2019). ‘False-positive’ self-reported psychotic experiences in the general population: an investigation of outcome, predictive factors and clinical relevance. Epidemiol Psychiatr Sci.

[60] Wright KB (2005). Researching Internet-based populations: advantages and disadvantages of online survey research, online questionnaire authoring software packages, and web survey services. J Comput-Mediat Comm.

[61] Louzon SA, Bossarte R, McCarthy JF, Katz IR (2016). Does suicidal ideation as measured by the PHQ-9 predict suicide among VA patients?. Psychiatr Serv.

[62] Penfold RB, Whiteside U, Johnson EE, Stewart CC, Oliver MM, Shortreed SM (2021). Utility of item 9 of the patient health questionnaire in the prospective identification of adolescents at risk of suicide attempt. Suicide Life Threat Behav.

[63] Uebelacker LA, German NM, Gaudiano BA, Miller IW (2011). Patient health questionnaire depression scale as a suicide screening instrument in depressed primary care patients: a cross-sectional study. Prim Care Companion CNS Disord.

[64] Hielscher E, DeVylder JE, Saha S, Connell M, Scott JG (2018). Why are psychotic experiences associated with self-injurious thoughts and behaviours? A systematic review and critical appraisal of potential confounding and mediating factors. Psychol Med.

