# Supplementary material for: Insomnia moderates the association between psychotic-like experiences and suicidal ideation in a non-clinical population: a network analysis
Source: Eur Arch Psychiatry Clin Neurosci. 2023 Jul 30;274(2):255–63. doi: 10.1007/s00406-023-01653-3 (PMC10914899; doi:10.1007/s00406-023-01653-3)
Supplement: Supplementary file 1 — Supplementary file1 (DOCX 1788 KB) [file 406_2023_1653_MOESM1_ESM.docx]

**Supplementary Appendix**

**Supplementary Figure 1.** Differences between all edge weights in participants with insomnia. (A) and between non-zero edge weights for connections between PLEs and suicidal ideation (D9) in higher resolution (B). Black boxes represent significant differences.

**A.**


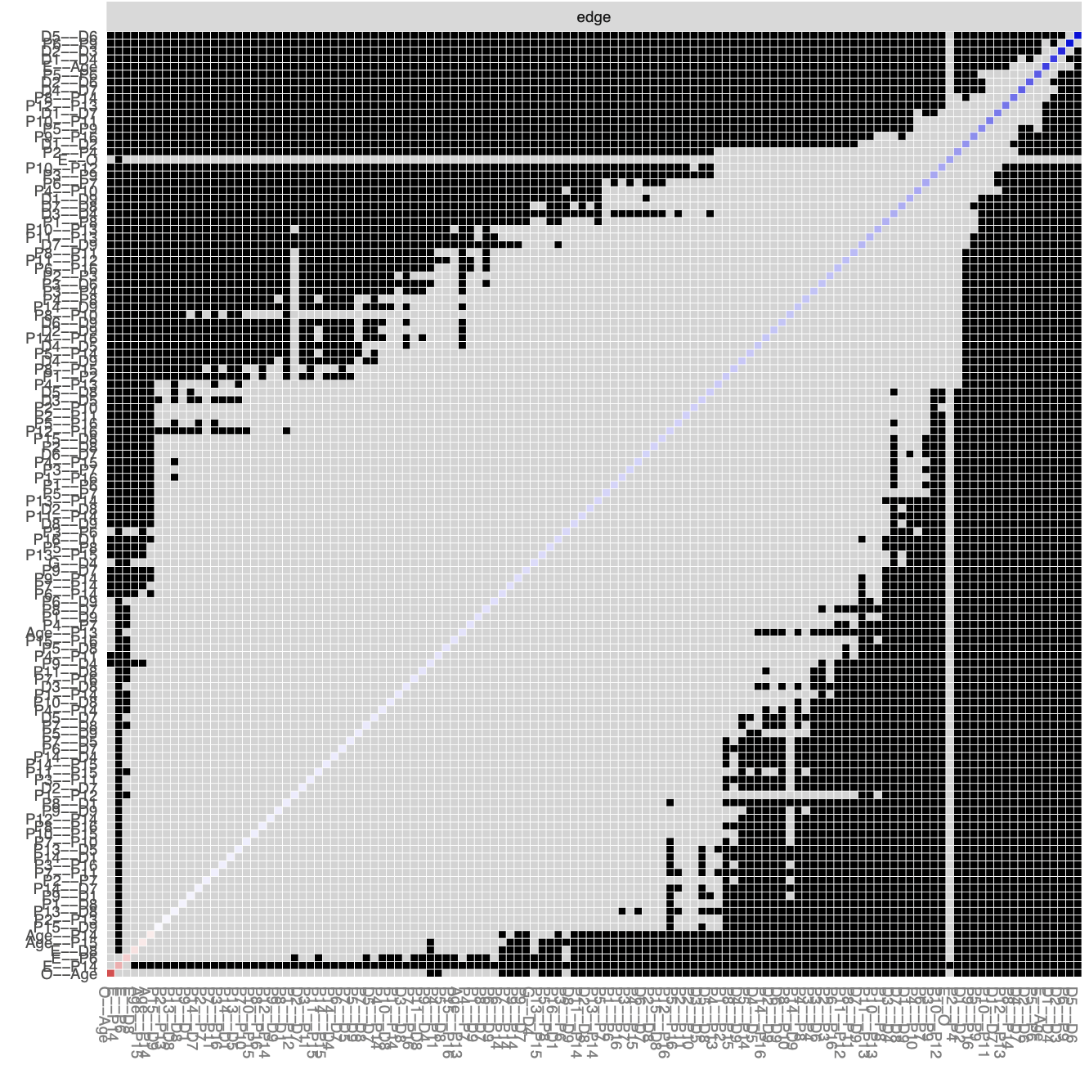


**B.**

|  | P1 – D9 | P5 – D9 | P6 – D9 | P9 – D9 | P14 – D9 |
| --- | --- | --- | --- | --- | --- |
| P1 – D9 | – |  |  |  |  |
| P5 – D9 |  | – |  |  |  |
| P6 – D9 |  |  | – |  |  |
| P9 – D9 |  |  |  | – |  |
| P14 – D9 |  |  |  |  | – |
| P15 – D9 |  |  |  |  |  |

**Supplementary Figure 2.** Differences between all edge weights in participants without insomnia. Black boxes represent significant differences.


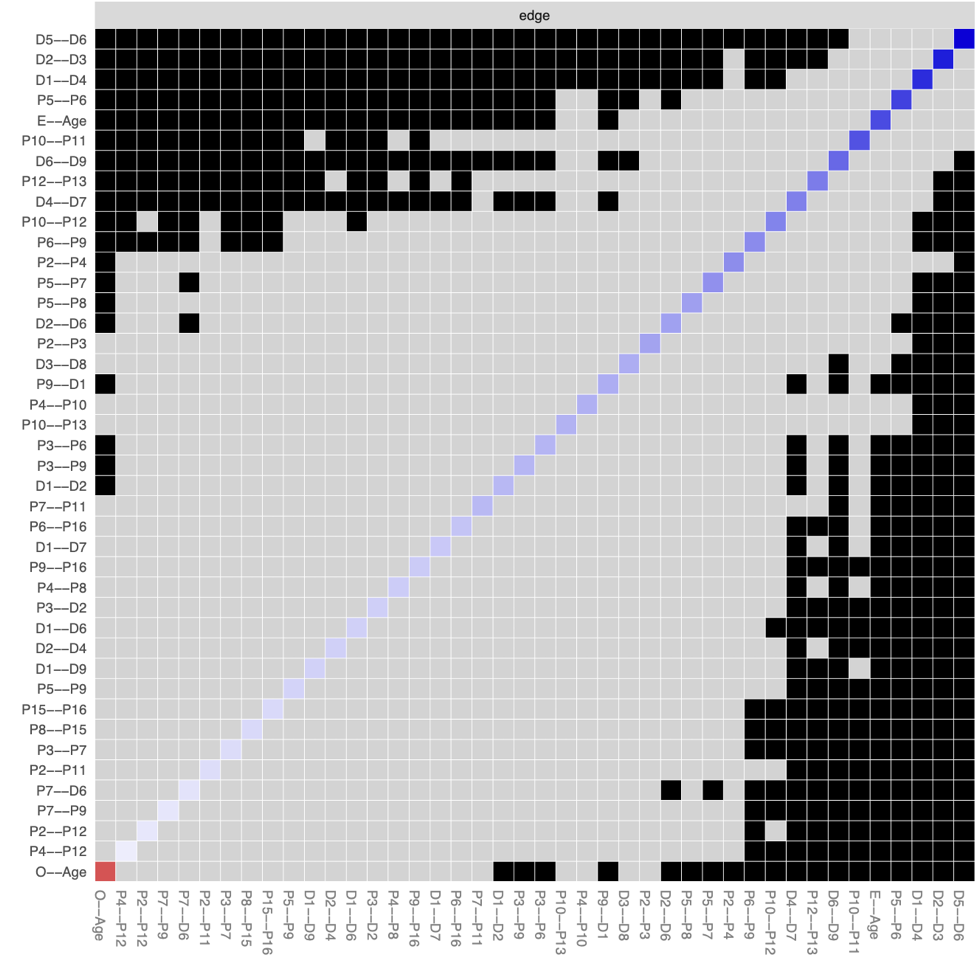


**Supplementary Figure 3.** Centrality values in participants with insomnia (A) and without insomnia (B).

**A.**


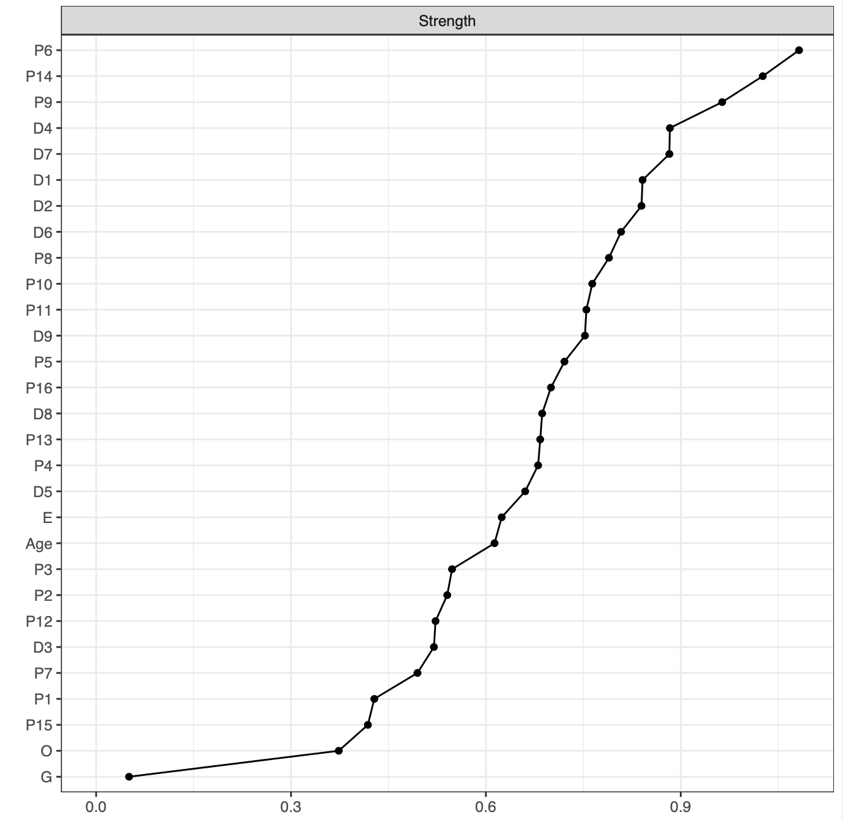


**B.**


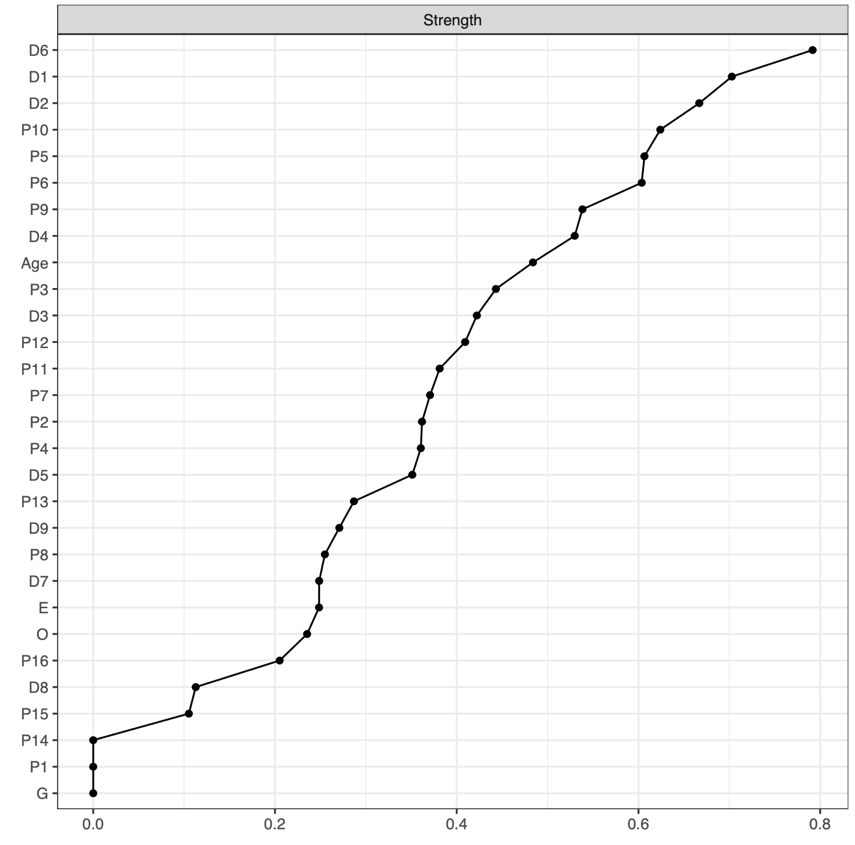


**Supplementary Figure 4.** Between-node differences in the strength centrality values among participants with insomnia (A) and those without insomnia (B).

**A.**


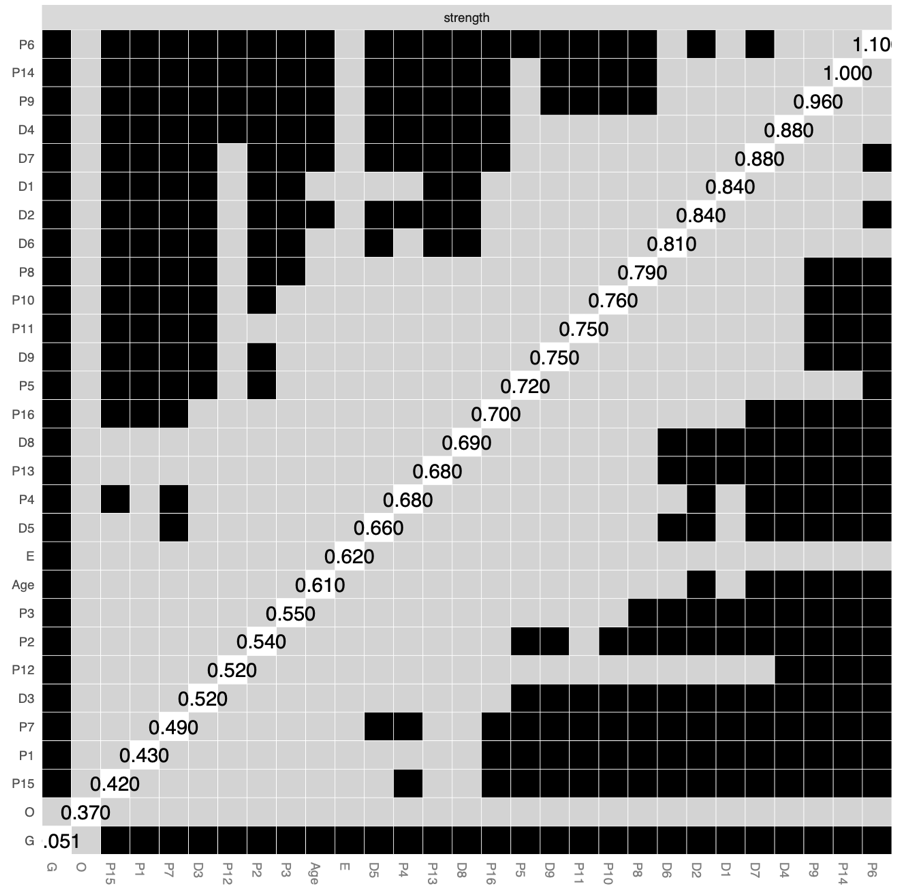


**B.**

**
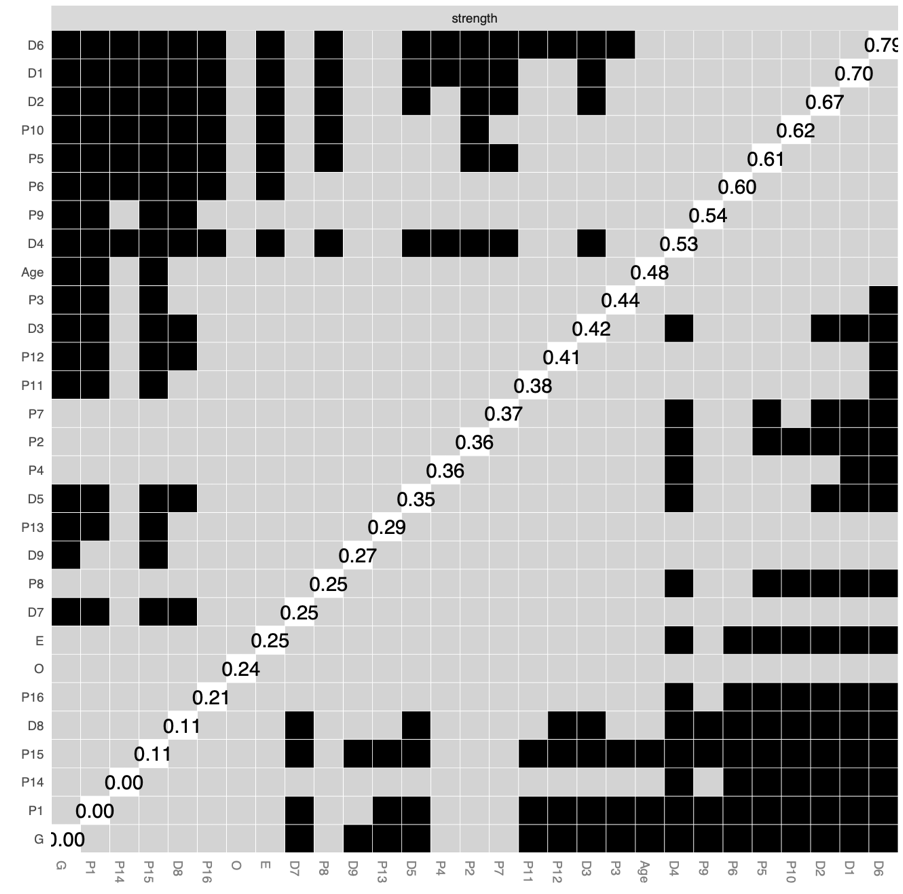
**

**Supplementary Figure 5.** Stability of the strength centrality index in the analysis of participants with insomnia (A) and the analysis of participants without insomnia (B). The red line shows the strength changes after removing various proportions of data.

**A.**


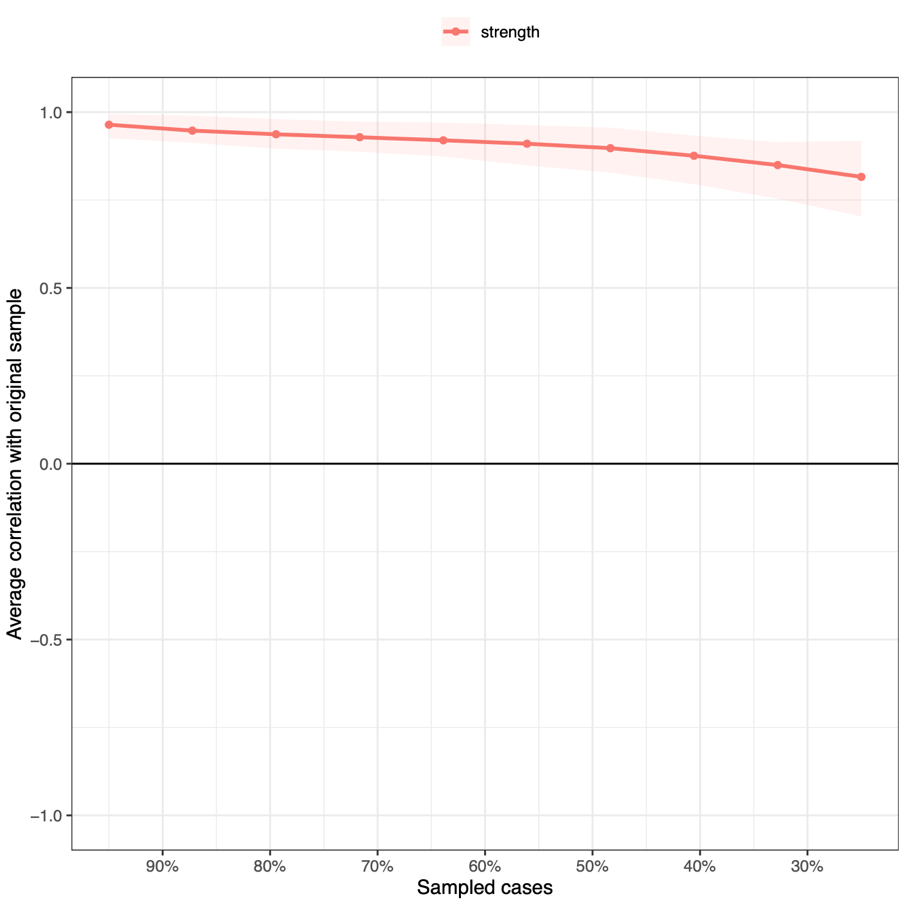


**B.**


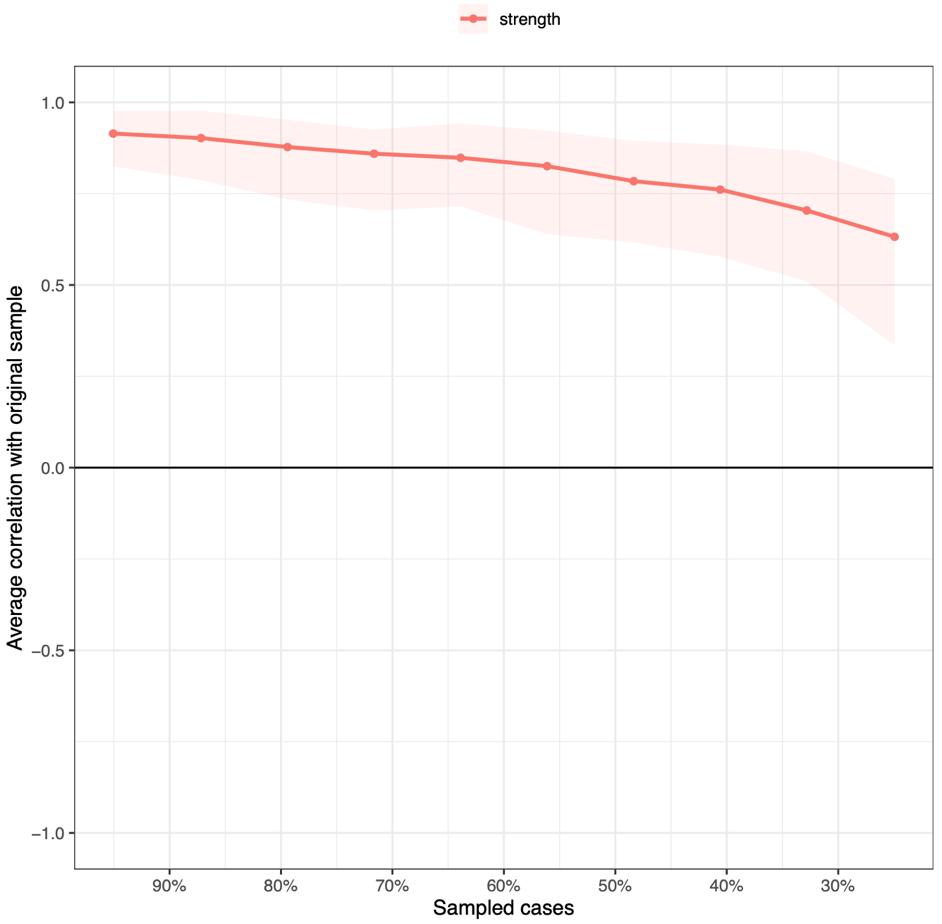


**Supplementary Figure 6.** Bootstrapped 95% confidence intervals of estimated edge weights in the analysis of participants with insomnia (A) and the analysis of participants without insomnia (B). The sample values are depicted with red lines. The bootstrapped 95% confidence intervals are shown within the grey area.

**A.**


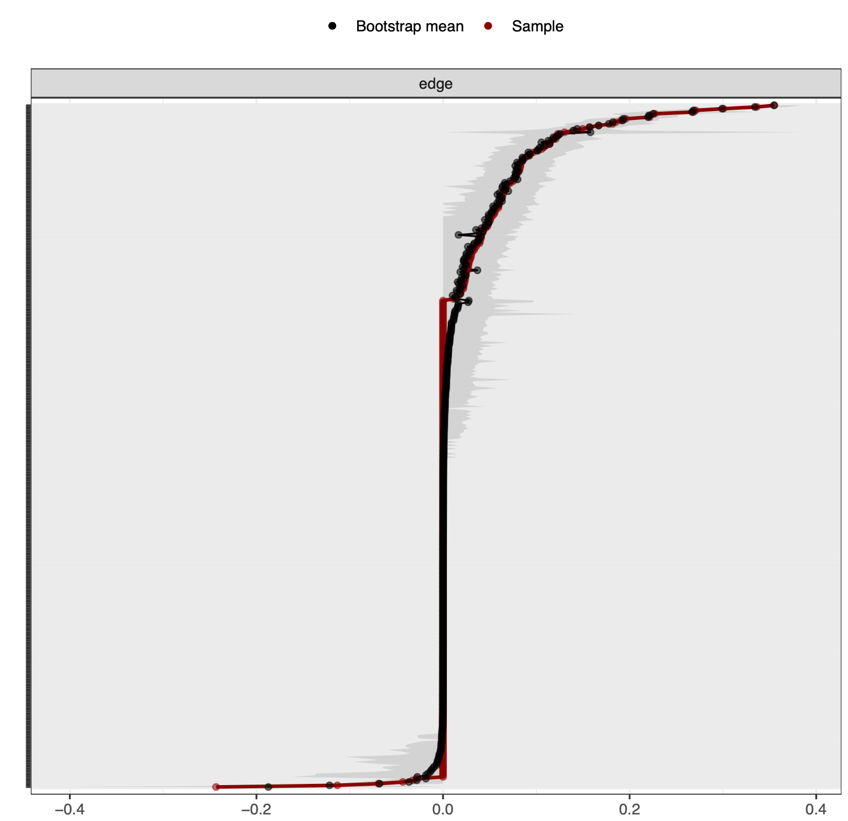


**B.**


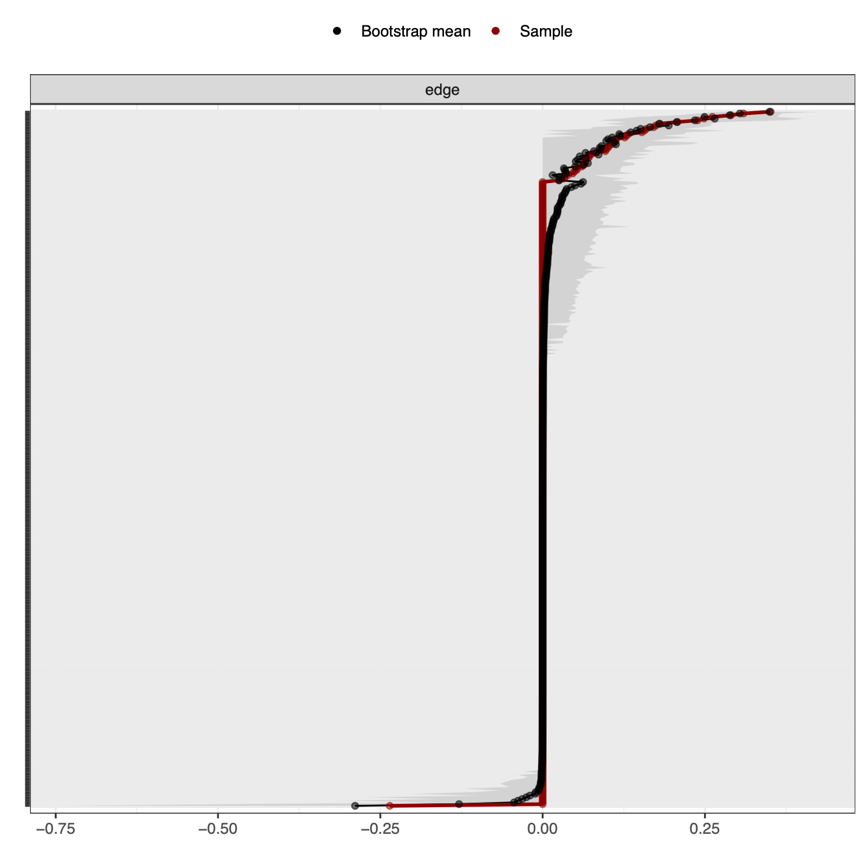


**Supplementary Table 1.** Items used to measure psychotic-like experiences.

| Questionnaire | Node name | Item |
| --- | --- | --- |
| RHS | P1 | “I hear voice speaking my thoughts aloud” |
|  | P15 | “I hear people call my name and find that nobody has done so” |
|  | P8 | “I see shadows and shapes when there is nothing there” |
| R-GPTS | P3 | “I spent time thinking about friends gossiping about me” |
|  | P5 | “People wanted me to feel threatened, so they stared at me” |
|  | P6 | “I was convinced there was a conspiracy about me” |
|  | P7 | “I was distressed by being persecuted” |
|  | P16 | “People have been dropping hints for me” |
| PQ16 | P2 | “When I look at a person, or look at myself in a mirror, I have seen the face change right before my eyes” |
|  | P4 | “I have heard things other people can’t hear like voices of people whispering or talking” |
|  | P9 | “I often feel that other have it in for me” |
|  | P10 | “I have seen things that other people apparently can’t see” |
|  | P11 | “I have had the sense that some person or force is around me, even though I could not see anyone” |
|  | P12 | “I sometimes see special meanings in advertisements, shop windows, or in the way things are arranged around me” |
|  | P13 | “I sometimes smell or taste things that other people can’t smell or taste” |
|  | P14 | “I often seem to live through events exactly as they happened before” |

Abbreviations: PQ16, the Prodromal Questionnaire 16 (Ising *et al.*, 2012); R-GPTS, the Revised Green Paranoid Thoughts Scale (Freeman *et al.*, 2021); RHS, the Revised Hallucination Scale (Gaweda and Kokoszka, 2011, Morrison *et al.*, 2000, 2002)

**Supplementary Table 2.** Edge weights in participants with insomnia.


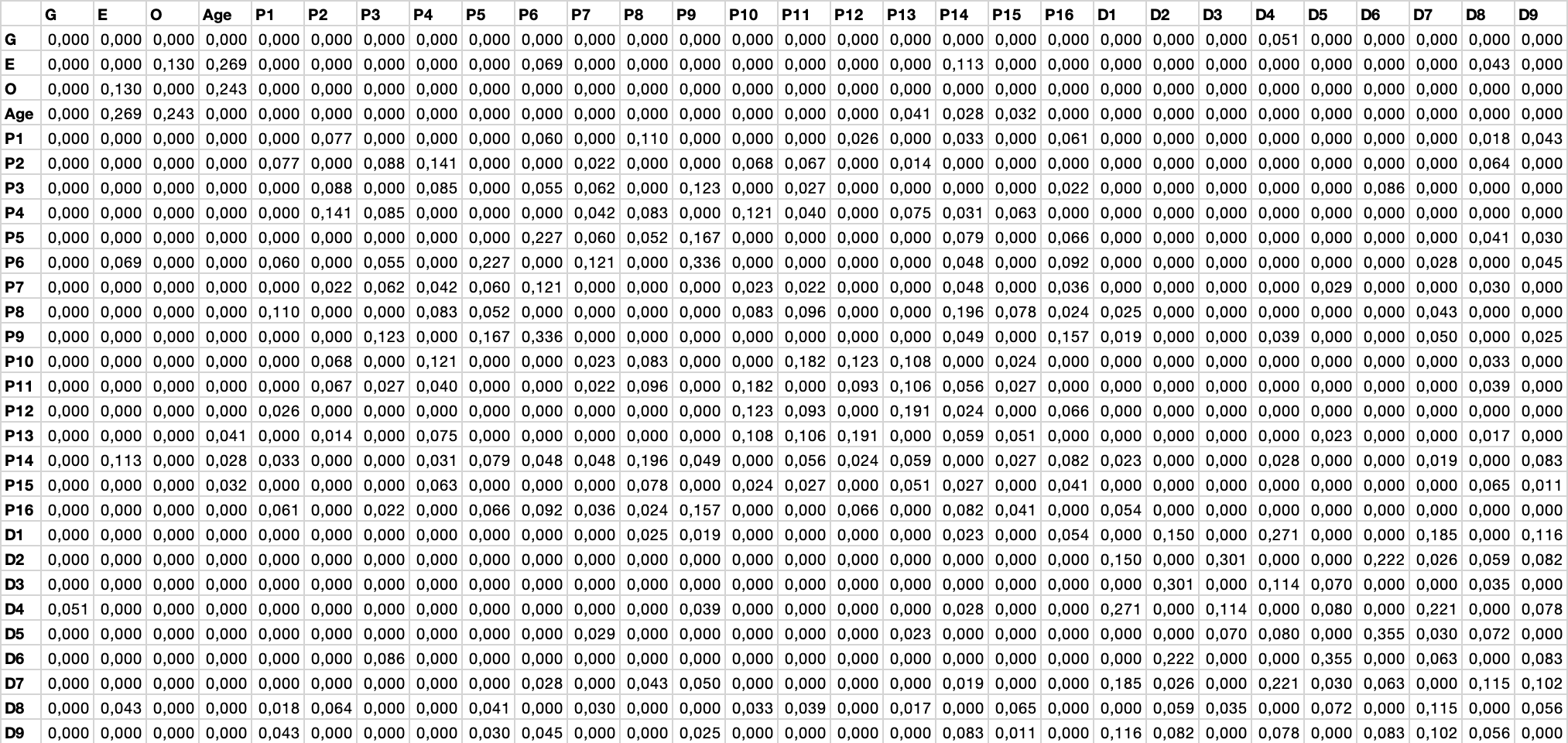


**Supplementary Table 3.** Edge weights in participants without insomnia.


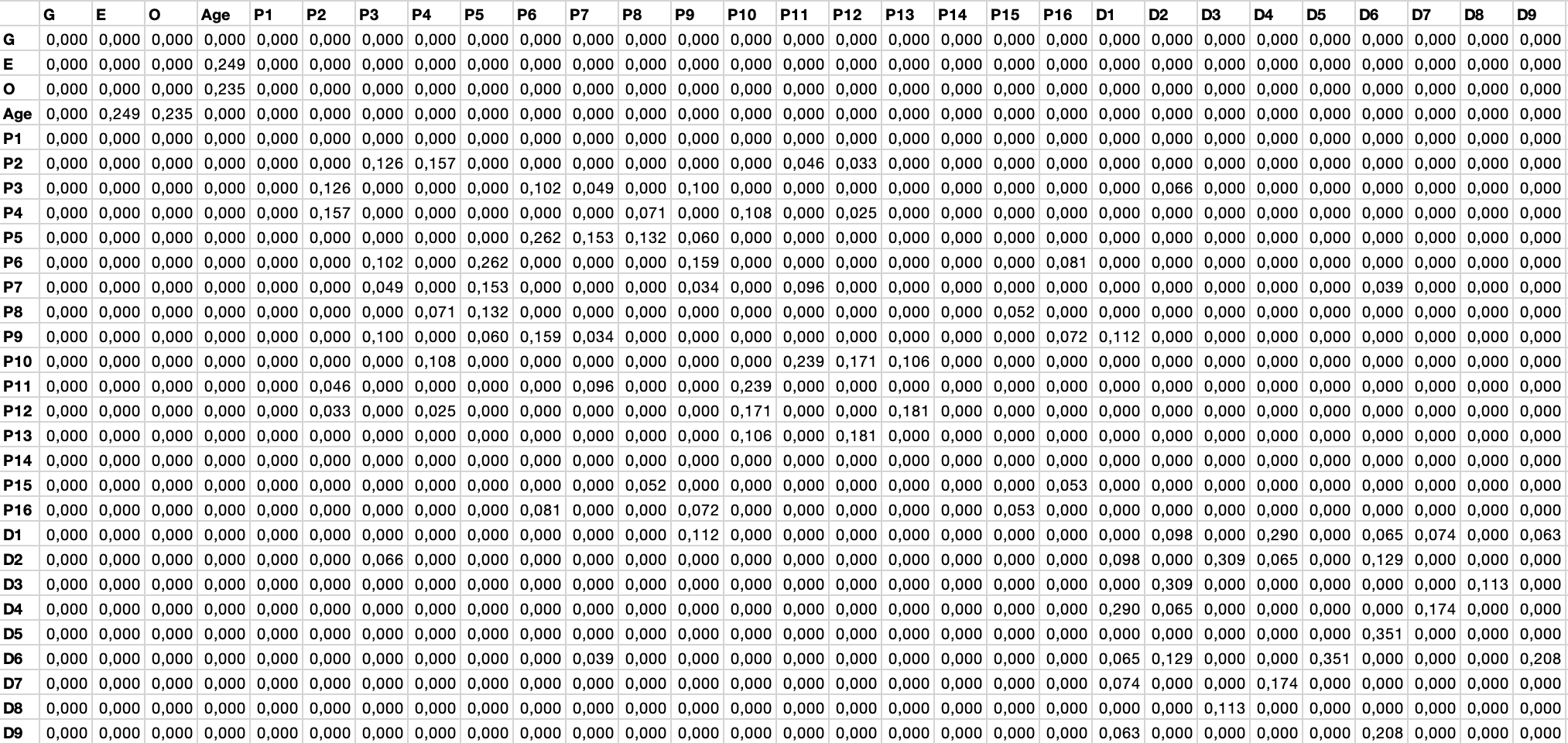


**Supplementary Table 4.** Node predictabilities.

| **Variable** | **Participants with insomnia** | **Participants without insomnia** |
| --- | --- | --- |
| G | 0.000 | 0.000 |
| E | 0.077 | 0.016 |
| O | 0.006 | 0.000 |
| Age | 0.068 | 0.066 |
| P1 | 0.133 | 0.000 |
| P2 | 0.161 | 0.123 |
| P3 | 0.204 | 0.193 |
| P4 | 0.192 | 0.134 |
| P5 | 0.341 | 0.276 |
| P6 | 0.452 | 0.267 |
| P7 | 0.183 | 0.155 |
| P8 | 0.261 | 0.135 |
| P9 | 0.451 | 0.244 |
| P10 | 0.24 | 0.249 |
| P11 | 0.249 | 0.203 |
| P12 | 0.179 | 0.202 |
| P13 | 0.208 | 0.142 |
| P14 | 0.322 | 0.052 |
| P15 | 0.126 | 0.036 |
| P16 | 0.266 | 0.127 |
| D1 | 0.376 | 0.352 |
| D2 | 0.358 | 0.328 |
| D3 | 0.237 | 0.211 |
| D4 | 0.384 | 0.300 |
| D5 | 0.299 | 0.228 |
| D6 | 0.363 | 0.387 |
| D7 | 0.368 | 0.159 |
| D8 | 0.200 | 0.078 |
| D9 | 0.288 | 0.134 |
| Mean | 0.241 | 0.165 |

**References**

**Freeman, D., Loe, B. S., Kingdon, D., Startup, H., Molodynski, A., Rosebrock, L., Brown, P., Sheaves, B., Waite, F. & Bird, J. C.** (2021). The revised Green et al., Paranoid Thoughts Scale (R-GPTS): psychometric properties, severity ranges, and clinical cut-offs. *Psychol Med* **51**, 244-253.

**Gaweda, L. & Kokoszka, A.** (2011). [Polish version of the Revised Hallucination Scale (RHS) by Morrison et al. Its factor analysis and the prevalence of hallucinatory-like experiences among healthy participants]. *Psychiatr Pol* **45**, 527-43.

**Ising, H. K., Veling, W., Loewy, R. L., Rietveld, M. W., Rietdijk, J., Dragt, S., Klaassen, R. M., Nieman, D. H., Wunderink, L., Linszen, D. H. & van der Gaag, M.** (2012). The validity of the 16-item version of the Prodromal Questionnaire (PQ-16) to screen for ultra high risk of developing psychosis in the general help-seeking population. *Schizophr Bull* **38**, 1288-96.

**Morrison, A. P., Wells, A. & Nothard, S.** (2000). Cognitive factors in predisposition to auditory and visual hallucinations. *Br J Clin Psychol* **39**, 67-78.

**Morrison, A. P., Wells, A. & Nothard, S.** (2002). Cognitive and emotional predictors of predisposition to hallucinations in non-patients. *Br J Clin Psychol* **41**, 259-70.
